# Preclinical biodistribution and dosimetry and human biodistribution comparing ^18^F-rhPSMA-7 and single isomer ^18^F-rhPSMA-7.3

**DOI:** 10.1186/s13550-021-00872-w

**Published:** 2022-02-04

**Authors:** Karina Knorr, So Won Oh, Markus Krönke, Alexander Wurzer, Calogero D’Alessandria, Michael Herz, Wolfgang Weber, Hans-Jürgen Wester, Matthias Eiber, Nahid Yusufi, Stephan Nekolla

**Affiliations:** 1 School of Medicine, Klinikum Rechts der Isar, Department of Nuclear Medicine, Technical University of Munich, Ismaninger Straße 22, 81675 Munich, Germany; 2 Department of Nuclear Medicine, Seoul National University Boramae Medical Center, Seoul, Korea; 3 School of Medicine, Klinikum Rechts der Isar, Department of Diagnostic and Interventional Radiology, Technical University of Munich, Munich, Germany; 4 Chair for Pharmaceutical Radiopharmacy, TUM, Garching, Germany

**Keywords:** ^18^F, Biodistribution, Dosimetry, PET/CT, PSMA, rhPSMA

## Abstract

**Background:**

Radiohybrid prostate-specific membrane antigen (rhPSMA) ligands such as ^18^F-rhPSMA-7 are a new class of theranostic agents in clinical development for prostate cancer. We compared preclinical dosimetry and human biodistribution of ^18^F-rhPSMA-7 with that of single diastereoisomer form, ^18^F-rhPSMA-7.3.

**Methods:**

Preclinical dosimetry was performed with SCID-mice sacrificed at multiple timepoints (10–300 min) post-injection of 25.6 ± 3.6 MBq ^18^F-rhPSMA-7 or 28.5 ± 4.8 MBq ^18^F-rhPSMA-7.3 (*n* = 3–6 mice per timepoint). Heart, lung, liver, spleen, pancreas, fat, stomach, small intestine, large intestine, kidney, muscle, bone, bladder, testicles, tail, and brain tissue were harvested, and urine and blood samples collected. Percentage of injected dose per gram was calculated. Absorbed doses were estimated with OLINDA/EXM 1.0.

^18^F-rhPSMA-7 (*n* = 47) and ^18^F-rhPSMA-7.3 (*n* = 33) PET/CT exams were used to estimate human biodistribution. Mean (range) injected activities were 324 (236–424) MBq versus 345 (235–420) MBq, and acquisition times were 84 (42–166) versus 76 (59–122) minutes for ^18^F-rhPSMA-7 versus ^18^F-rhPSMA-7.3, respectively. SUV_mean_ was determined for background (gluteal muscle), normal organs (salivary glands, blood pool, lung, liver, spleen, pancreas, duodenum, kidney, bladder, bone) and up to three representative tumour lesions. Qualitative analyses assessed image quality, non-specific blood pool activity, and background uptake in bone/marrow using 3/4-point scales.

**Results:**

Preclinical dosimetry revealed that at 3.5 h and 1 h bladder voiding intervals, the extrapolated total effective doses were 26.6 and 12.2 µSv/MBq for ^18^F-rhPSMA-7 and 21.7 and 12.8 µSv/MBq for ^18^F-rhPSMA-7.3 respectively.

Human biodistribution of both agents was typical of other PSMA-ligands and broadly similar to each other; SUV_mean_ were 16.9 versus 16.2 (parotid gland), 19.6 versus 19.9 (submandibular gland), 2.0 versus 1.9 (blood pool, *p* < 0.005), 0.7 versus 0.7 (lungs), 7.0 versus 7.3 (liver), 9.1 versus 8.4 (spleen), 32.4 versus 35.7 (kidney), 2.5 versus 2.8 (pancreas), 10.9 versus 11.0 (duodenum), 1.1 versus 1.3 (bone) and 4.6 versus 2.0 (bladder; *p* < 0.001) for ^18^F-rhPSMA-7 versus ^18^F-rhPSMA-7.3, respectively. Tumour SUV_mean_ was higher for ^18^F-rhPSMA-7.3 (32.5 ± 42.7, *n* = 63 lesions) than for ^18^F-rhPSMA-7 (20.0 ± 20.2, *n* = 89 lesions).

**Conclusions:**

Radiation dosimetry is favourable for both agents. Radiation exposure, assuming a 1 h voiding interval, is less than 5 mSv after injection of 370 MBq. ^18^F-rhPSMA-7.3 showed significantly lower bladder uptake, and a higher uptake trend in tumours compared with ^18^F-rhPSMA-7.

**Supplementary Information:**

The online version contains supplementary material available at 10.1186/s13550-021-00872-w.

## Background

Prostate-specific membrane antigen (PSMA) is overexpressed in nearly all primary prostate cancer [1], and PSMA expression further increases in de-differentiated, metastatic, or hormone-refractory disease [2, 3]. In recent years, PSMA targeting ligands have been investigated for molecular imaging and radioligand therapy of prostate cancer. Recent developments increasingly focus on ^18^F-based, rather than ^68^ Ga-based PSMA ligands for imaging given the advantages of ^18^F-based agents in terms of availability, ease of production, and image resolution.

Several ^18^F-based PSMA ligands have been developed with different kinetic profiles. The kidneys and the urinary tracts are critical organs in the development of novel PSMA ligands as intense urinary retention may interfere with accurate evaluation of the prostate and adjacent area. ^18^F-labelled, urea-based inhibitors of PSMA such as ^18^F-DCFBC and ^18^F-DCFPyL demonstrated diagnostic capabilities in clinical trials [4, 5], but they still have shortcomings due to urinary excretion as their limitation to a diagnostic use. ^18^F-PSMA-1007 was developed aiming for similar structure, biodistribution and tumour uptake compared to PSMA-617 which is currently used for radioligand therapy. ^18^F-PSMA-1007 is predominantly excreted via the hepatobiliary tract and has very low urinary retention but its optimal imaging timepoint (120 min) is suboptimal for routine clinical practice [6].

Radiohybrid PSMA (rhPSMA) ligands are a new class of fully theranostic agents that allow fast ^18^F synthesis and labelling with radiometals. They are silicon-fluoride-acceptor (SiFA)–conjugated radiopharmaceuticals that are labeled by isotopic exchange. One example, ^18^F-rhPSMA-7, has been shown to have a biodistribution typical of other established PSMA ligands [7] and its clinical performance for imaging patients with prostate cancer has been reported previously [8, 9]. ^18^F-rhPSMA-7 is comprised of four diastereoisomers (rhPSMA-7.1, rhPSMA-7.2, rhPSMA-7.3 and rhPSMA-7.4), of which rhPSMA-7.3 exhibits the most promising targeting characteristics for clinical translation and has been selected for further development based in preclinical results [10].

Here, we analyzed the preclinical biodistribution and dosimetry of ^18^F-rhPSMA-7 and ^18^F-rhPSMA-7.3 at different timepoints after a single intravenous administration in mice. We further assessed biodistribution and image quality of clinical ^18^F-rhPSMA-7 and ^18^F-rhPSMA-7.3 PET scans in patients with histopathologically proven prostate cancer.

## Methods

### Synthesis of ^18^F-rhPSMA-7 and ^18^F-rhPSMA-7.3

The rhPSMA-7 and rhPSMA-7.3 peptide precursors were labelled with ^18^F and dissolved in phosphate buffered saline solution as previously described [11]. The ^18^F-labeling of rhPSMA-7 and rhPSMA-7.3 was performed in a fully automated, Good Manufacturing Practice-compliant procedure using a GRP™ synthesis module (Scintomics, Fürstenfeldbruck, Germany).

### Preclinical biodistribution and dosimetry

Severe combined immunodeficiency (SCID) mice were supplied by Charles River Laboratories. (Freiburg, Germany). The preclinical evaluation study was performed in accordance with the German Animal Welfare Act (Deutsches Tierschutzgesetz, approval #55.2-1-54-2532-216-2015). Male mice (≥ 6 weeks old) were used for the study after reaching sexual maturation.

The biodistribution study was performed at multiple timepoints; 10, 20, 40, 60, 120 and 180 min post-injection of ^18^F-rhPSMA-7, and at 10, 60, 120, 180 and 300 min post-injection of ^18^F-rhPSMA-7.3. Based on initial experiments exhibiting prolonged renal uptake for ^18^F-rhPSMA-7.3, a late timepoint (300 min) was applied for the final experiments. At each timepoint, 3–6 mice were injected intravenously in the tail vein with a mean 25.6 ± 3.6 MBq of ^18^F-rhPSMA-7 and 28.5 ± 4.8 MBq of ^18^F-rhPSMA-7.3, respectively. For the biodistribution study, the mice were dissected and samples collected from urine, blood, heart, lung, spleen, pancreas, liver, stomach (emptied), small intestine (emptied), large intestine (emptied), kidneys, bladder, testis, fat, muscle (partial, femoral), femur, tail and brain. An automatic gamma counter (PerkinElmer-Wallac, Waltham, USA) was used to measure count rate and the percentages of the injected dose (%ID and %ID/g) were calculated.

Of note, to achieve a similar number of timepoints for both calculations, the ^18^F-rhPSMA-7 10 min and 20 min timepoints were interpolated to a 15 min timepoint. The time-integral of activity for the accumulation in the investigated source organs (AUCs) were generated both with numerical integration and physical decay [12].

To extrapolate from preclinical data to human dosimetry, linear scaling of %ID from the mice by the ratio of the organ weights and total body weights of phantoms compared to humans was necessary [13]. Normal-organ radiation doses were estimated for the 70 kg standard adult anatomic model using time-dependent organ activity concentrations in %ID/g and total-body activities measured in the biodistribution studies in mice. Tissue activity concentrations in mice were converted to tissue fractional activities in the 70 kg standard adult using the relative fractional organ masses in the standard adult and the standard 25 g mouse. Time-dependent total-body activity was fit to an exponential function and the difference between the injected activity and the total-body activity was assumed to be excreted via the urine because activity concentrations in the liver and gastrointestinal tract were low at all timepoints studied.

Organ residence time was calculated by numerical integration using the trapezoidal rule and the rest-of-body. ^18^F residence times were calculated as the difference between the total-body residence time and the sum of the organ and urine residence times. The bladder contents residence time was estimated using the dynamic voiding model in the OLINDA/EXM 1.0 dosimetry software (Vanderbilt University, Nashville, TN, USA). Finally, the standard adult mean absorbed dose to organs (in µGy/MBq) and total effective dose (in µSv/MBq) were calculated using OLINDA/EXM 1.0 [14].

### Human biodistribution

#### Patients

Data from patients with histopathologically proven prostate cancer who underwent a clinically indicated PET/CT with ^18^F-rhPSMA-7 or ^18^F-rhPSMA-7.3 between October 2017 and November 2018 were retrospectively analyzed. ^18^F-rhPSMA-7 PET/CT was performed in 47 patients (mean [range] age: 69.8 [52–80] years) with a mean injected activity of 324 (range, 236–424) MBq and mean acquisition time of 84 (range, 42–166) min. ^18^F-rhPSMA-7.3 PET/CT was performed in 33 patients (mean [range] age: 70.8 [57–85] years) with a mean injected activity of 345 (range, 235–420) MBq and mean acquisition time of 76 (range, 59–122) min. The mean prostate-specific antigen level at the time of imaging was 42.9 ng/mL (median range, 0–1459 ng/mL) for ^18^F-rhPSMA-7 and 20.0 ng/mL (range, 0–202 ng/mL) for ^18^F-rhPSMA-7.3, respectively. Patients received an injection of 20 mg of furosemide at the time of tracer application. A comparison of the disease stages and primary Gleason Scores of both cohorts are presented in Additional file 1: Table S5.

All patients gave written informed consent for the original procedure. All reported investigations were conducted in accordance with the Helsinki Declaration and with national regulations. This retrospective analysis was approved by the Local Ethics Committee (permits 290/18S and 99/19S) and the need for patient consent was waived. The administration of ^18^F-rhPSMA-7 and ^18^F-rhPSMA-7.3 was in accordance with The German Medicinal Products Act (AMG §13 2b) and the responsible regulatory body (Government of Oberbayern).

#### PET/CT imaging

All the PET/CT scans were obtained from the skull base to mid-thigh using a Biograph mCT Flow scanner (Siemens Medical Solutions, Erlangen, Germany). PET scanning was operated in 3D mode with an acquisition time of 1.1 mm/s in continuous table movement, and a diagnostic CT scan (240 mAs, 120 kV, 5 mm slice thickness) was acquired in the portal venous phase 80 s after the intravenous injection of an iodinated contrast agent (Imeron 400, Bracco Imaging Deutschland GmbH, Konstanz, Germany). Reconstruction of the PET images was performed based on iterative algorithms with an ordered-subsets expectation maximization (4 iterations, 8 subsets) followed by a post-reconstruction smoothing Gaussian filter (5 mm full width at one-half maximum).

#### Image analysis

For both quantitative and qualitative analyses PET datasets (non-Time-of-Flight/non-True X) were used. The maximum standardized uptake value (SUV_max_) and the mean standardized uptake value (SUV_mean_) with an isocontour of 50% of the SUV_max_ were determined applying circular volumes of interest (VOIs) using OsiriX MD® 11.0.2 (Pixmeo SARL, Geneva, Switzerland).

The circular VOIs were placed over normal organs; parotid gland, submandibular gland, mediastinal aortic arch (blood pool), lungs, liver, spleen, pancreas, duodenum, kidneys, bladder, sacral promontory, and background (gluteus maximus muscle). Up to 3 lesions per patient were analyzed in decreasing order of SUV_max_. Organ/tumour to background ratios (ratio-SUV_mean_, ratio-SUV_max_) were calculated.

To evaluate overall image quality, non-specific blood pool activity and background uptake in bone/marrow was analysed using 3- or 4-point scales as previously described [7]. All the analyses were performed by a board-certified nuclear medicine physician.

### Statistical analysis

Prior to analysis, the Kolmogorov–Smirnov test was used to assess the normality of the data distribution. The independent Student t-test was performed to compare means between groups for the normal parameters. The Mann–Whitney U test was conducted for the non-normal parameters. The Chi-square test or the Fisher's exact test was adopted to compare differences among groups for the analyses of ordinal variables. Data were expressed as mean ± standard deviation (SD) for continuous variables and frequencies (percentages, %) for categorical variables, respectively. All statistical analyses were performed using the IBM SPSS Statistics version 25 (IBM Inc. Armonk, NY, USA) and R version 3.5.2 (http://www.r-project.org). *P* values less than 0.05 were considered statistically significant.

## Results

### Preclinical biodistribution and dosimetry

Significant accumulation of radioactivity was observed (i.e. source organs) in the kidney, spleen, lung, liver and heart for both ^18^F-rhPSMA-7 and ^18^F-rhPSMA-7.3. The activity distributed to individual organs is presented for the source organs and whole organs in Additional file 1: Table S1 and S2, respectively. The AUCs in the source organs are summarized in Table 1, and only results calculated from the AUCs with extrapolation and physical decay were used in the following analyses. Radioactivity distribution of both ^18^F-rhPSMA-7 and ^18^F-rhPSMA-7.3 was found to be the highest in the kidneys at most timepoints. With respect to activity accumulation and clearance, a rapid clearance from blood and clearance to urine but relatively slow build-up in the kidneys was present for both ^18^F-rhPSMA-7 and ^18^F-rhPSMA-7.3.

**Table 1:** Time-integral of activity for accumulation in significant source organs (AUCs)

	AUC (%IA/g) organ	Heart	Lung	Liver	Spleen	Kidney
^18^F-rhPSMA-7	No extrapolation	0.17 ± 0.01	0.84 ± 0.04	0.78 ± 0.05	1.93 ± 0.13	6.39 ± 0.65
	Extrapolation + physical decay	0.18 ± 0.01	0.9 ± 0.04	0.87 ± 0.06	2.1 ± 0.13	10.12 ± 0.75
^18^F-rhPSMA-7.3	No extrapolation	0.29 ± 0.03	1.35 ± 0.12	1.25 ± 0.08	2.05 ± 0.2	8.99 ± 0.64
	Extrapolation + physical decay	0.3 ± 0.03	1.38 ± 0.12	1.33 ± 0.08	2.08 ± 0.2	10.8 ± 0.74

AUC: area under the curve; %IA: %injected activity. AUC is presented ± standard deviation

Extrapolating the preclinical dosimetry data to humans revealed that at 1 h and 3.5 h bladder voiding intervals, the total effective doses for humans are 12.2 and 26.6 µSv/MBq for ^18^F-rhPSMA-7, and 12.8 and 21.7 µSv/MBq for ^18^F-rhPSMA-7.3, respectively (Table 2). The absorbed doses for the individual organs at 1 h and 3.5 h bladder voiding intervals are provided in Additional file 1: Table S3. On the individual organ level, the kidneys were among the organs receiving the highest absorbed doses for both ^18^F-rhPSMA-7 and ^18^F-rhPSMA-7.3. For both bladder voiding intervals, the absorbed dose in the kidneys was slightly higher with ^18^F-rhPSMA-7.3 (71.60 and 71.80 µGy/MBq at 1 h and 3.5 h, respectively) than with ^18^F-rhPSMA-7 (64.70 and 65.10 µGy/MBq at 1 h and 3.5 h, respectively; Additional file 1: Table S3).

**Table 2:** Dosimetry results using 1 h and 3.5 h voiding interval

Voiding interval	^18^F-rhPSMA-7	^18^F-rhPSMA-7.3
	Effective dose (µSv/MBq)	Effective dose (µSv/MBq)
1 h	12.2	12.8
3.5 h	26.6	21.7

### Human biodistribution in normal organs and tumours

**Fig. 1:**
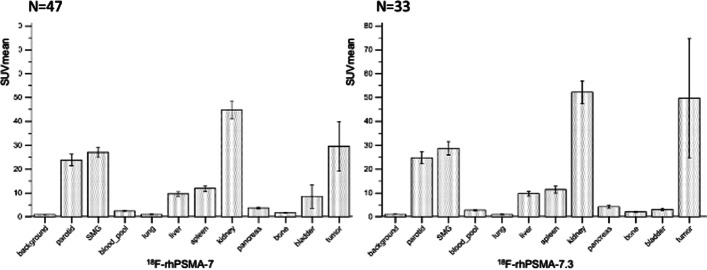
Human biodistribution in normal organs and tumours of ^18^F-rhPMSA-7 (A) and ^18^F-rhPSMA-7.3 using SUVmean. SMG: submandibular gland

Quantitative normal organ biodistribution was very similar for ^18^F-rhPSMA-7 and ^18^F-rhPSMA-7.3 (Fig. 1, Table 3). SUV_mean_ were 16.9 versus 16.2 (parotid gland), 19.6 versus 19.9 (submandibular gland), 2.0 versus 1.9 (blood pool, *p* < 0.005), 0.7 versus 0.7 (lungs), 7.0 versus 7.3 (liver), 9.1 versus 8.4 (spleen), 32.4 versus 35.7 (kidney), 2.5 versus 2.8 (pancreas), 10.9 versus 11.0 (duodenum), 1.1 versus 1.3 (non-diseased bone, *p* = 0.005) and 4.6 versus 2.0 (bladder; *p* < 0.001) for ^18^F-rhPSMA-7 versus ^18^F-rhPSMA-7.3, respectively. Relative to the other organs, the kidneys exhibited particularly high uptake values of both rhPSMA ligands, but radiotracer retention in the urinary bladder was comparatively low. Moreover, the mean bladder SUV_mean_ and ratio-SUV_mean_ were significantly lower for ^18^F-rhPSMA-7.3 than for ^18^F-rhPSMA-7 (*p* < 0.001; Table 3). Qualitative analyses of image quality revealed no significant differences between ^18^F-rhPSMA-7 and ^18^F-rhPSMA-7.3 PET (Additional file 1: Table S4).

**Table 3:** Comparisons of the individual organ uptake and tumour uptake in human biodistribution

	SUV_mean_			ratio-SUV_mean_		
	^18^F-rhPSMA-7 (*n* = 47)	^18^F-rhPSMA-7.3 (*n* = 33)	*p*-value^*^	^18^F-rhPSMA-7 (*n* = 47)	^18^F-rhPSMA-7.3 (*n* = 33)	*p-*value^*^
Background	0.63 ± 0.17	0.66 ± 0.13	0.0655^†^	–	–	–
Parotid gland	16.94 ± 5.98	16.19 ± 4.42	0.5494	28.31 ± 11.15	25.46 ± 8.78	0.2296
Submandibular gland	19.62 ± 5.32	19.86 ± 5.45	0.8540^†^	33.29 ± 12.34	31.00 ± 10.48	0.388
Blood pool	2.00 ± 2.26	1.85 ± 0.32	**0.0282**^†^	3.18 ± 2.81	2.88 ± 0.69	0.6007^†^
Lungs	0.68 ± 0.32	0.68 ± 0.19	0.5120	1.15 ± 0.63	1.04 ± 0.28	0.9492^†^
Liver	6.98 ± 2.31	7.29 ± 2.24	0.5561^†^	11.95 ± 6.12	11.39 ± 4.20	0.9571^†^
Spleen	9.14 ± 3.24	8.44 ± 3.23	0.3382^†^	15.12 ± 6.26	13.28 ± 5.69	0.1834
Kidneys	32.38 ± 9.33	35.74 ± 9.72	0.1953^†^	55.17 ± 22.69	56.06 ± 18.15	0.8534
Pancreas	2.54 ± 0.87	2.84 ± 0.98	0.1772^†^	4.27 ± 1.82	4.36 ± 1.47	0.5348^†^
Duodenum	10.94 ± 4.22	10.96 ± 4.66	0.9813	18.36 ± 7.64	17.09 ± 7.88	0.4006^†^
Bone	1.10 ± 0.33	1.34 ± 0.41	**0.0050**	1.81 ± 0.56	2.08 ± 0.71	0.0615
Bladder	4.59 ± 5.29	2.00 ± 0.78	**0.0008**	7.35 ± 7.79	3.09 ± 1.19	**0.0002**^†^
Tumour	20.03 ± 20.23	32.54 ± 42.71	0.0711^†^	35.97 ± 45.54	50.85 ± 68.65	0.1462^†^

Bold indicate that this are significant *p*-values (below 0.05)

SUV_mean_: mean standardized uptake value; ratio-SUV_mean_: organ/tumour-to-background ratios with background being gluteus maximus muscle

*Results of group comparisons from independent t-test or Mann–Whitney U test

^†^Normal distribution

Tumour uptake was analyzed in 89 lesions (26 primary tumours/local recurrences, 23 bone, 38 lymph node and 2 visceral metastases) and 63 lesions (14 primary tumours/local recurrences, 30 bone, 18 lymph node and 1 visceral metastases) for ^18^F-rhPSMA-7 and ^18^F-rhPSMA-7.3, respectively. Mean tumour SUV_mean_ (20.0 ± 20.2 for ^18^F-rhPSMA-7 vs. 32.5 ± 42.7 for ^18^F-rhPSMA-7.3) and mean ratio-SUV_mean_ (36.0 ± 45.5 for ^18^F-rhPSMA-7 vs. 50.9 ± 68.7 for ^18^F-rhPSMA-7.3) showed a trend towards higher tumour uptake values for ^18^F-rhPSMA-7.3 but without statistical significance (*p* > 0.05, Fig. 1, Table 3).

**Fig. 2:**
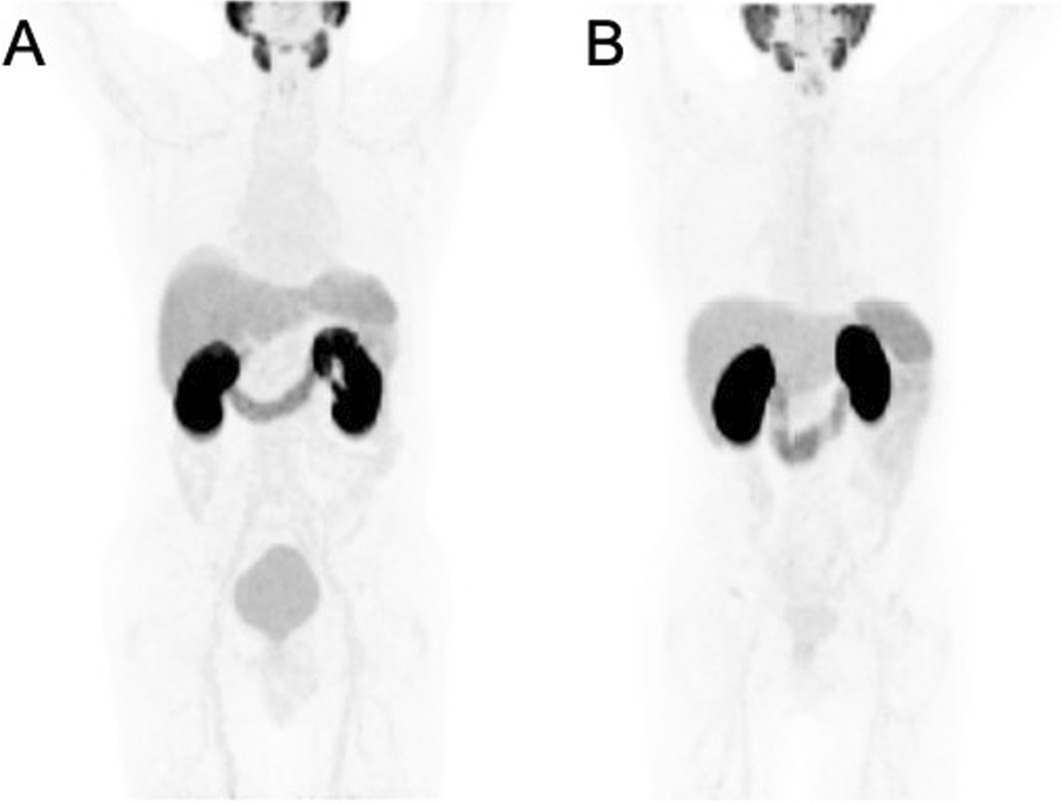
Maximum intensity projection images of patients with normal biodistribution of ^18^F-rhPSMA-7 (**A**) and ^18^F-rhPSMA-7.3 (**B**), respectively

Figure 2 demonstrates the maximum intensity projection images of patients with normal biodistribution of ^18^F-rhPSMA-7 and ^18^F-rhPSMA-7.3. Additional file 1: Figure S1 demonstrates the maximum intensity projection images and PET/CT fused images of two patients with early biochemical recurrence who have undergone ^18^F-rhPSMA-7 and ^18^F-rhPSMA-7.3 PET/CT, respectively.

## Discussion

Here, we conducted a series of preclinical and human biodistribution and dosimetry studies to compare the profiles of two lead rhPSMA ligands for prostate cancer imaging. The results of the preclinical elements of this study indicate that the biodistribution and dosimetry profiles for both ^18^F-rhPSMA-7 and its single diastereoisomer form, ^18^F-rhPSMA-7.3, are favourable for prostate cancer imaging. The human clinical biodistribution results confirm and extend the preclinical data to indicate that while the profiles of ^18^F-rhPSMA-7 and ^18^F-rhPSMA-7.3 are similar, the significantly lower bladder uptake, and the trend towards higher uptake in tumours with ^18^F-rhPSMA-7.3 suggest that it is the more promising agent for further clinical translation.

The AUCs in the mouse dosimetry studies showed that ^18^F-rhPSMA-7.3 exhibited appropriate profiles for human study. Compared with ^18^F-rhPSMA-7, kidney uptakes of ^18^F-rhPSMA-7.3 were higher, but its accumulation speed was relatively slow. Thus, dosimetry studies of ^18^F-rhPSMA-7.3 were planned to be performed at a late timepoint (300 min) to acknowledge this slow build-up in the kidneys. This kinetic profile could be helpful for PSMA ligand imaging. Since urinary excretion of the radiotracer is slow, excretion may not contribute to the kidney uptakes at early imaging timepoints, which in turn leads to lower bladder radioactivity levels and lower levels of background.

While direct conversion of preclinical dosimetry studies using mice into human data will not truly model the human profile, it is reasonable to assume that kinetic profiles are similar in humans. In our earlier work with ^18^F-rhPSMA-7, we recommended an early imaging timepoint (50–70 min) for ^18^F-rhPSMA-7 as lower tracer retention in the urinary bladder is an important feature for PSMA ligand PET imaging [7] and it is expected that PET/CT with ^18^F-rhPSMA-7.3 could also achieve good image quality at early imaging timepoints, especially given the significantly lower bladder SUV that we observe here.

The extrapolated effective doses for humans calculated from the preclinical dosimetry show estimated effective doses of both rhPSMA ligands that are comparable with those of other ^18^F-based PSMA ligands. The estimated effective doses using 1 h voiding interval were 12.2 µSv/MBq for ^18^F-rhPSMA-7 and 12.8 µSv/MBq for ^18^F-rhPSMA-7.3, respectively, leading to an effective dose of less than 5 mSv after injection of 370 MBq of either rhPSMA ligand. Radiation dosimetry studies revealed a mean effective dose of 19.9 µSv/MBq for ^18^F-DCFBC [4], 13.9 µSv/MBq for ^18^F-DCFPyL [5], and 22.0 µSv/MBq for ^18^F-PSMA-1007 [6]. ^18^F-rhPSMA-7 and ^18^F-PSMA-7.3 also provide favourable effective doses in comparison with ^18^F-FDG, which has a reported effective dose of 19.0 µSv/MBq [15]. In the present study the kidneys received among the highest doses with both rhPSMA ligands. This is in line with data previously reported for ^18^F-rhPSMA-7.3 in healthy volunteers [16]. Tolvanen et al. reported the kidneys to be one of the organs with the highest mean absorbed doses (172 µGy/MBq at a 3.5 h voiding window) [16]. All PSMA ligands share similar physiological distribution; organ absorbed doses are known to differ minimally among various PSMA ligands [6] and the data for ^18^F-rhPSMA-7.3 appear comparable with other agents.

Our clinical data demonstrate that, in humans, the normal biodistribution of ^18^F-rhPSMA-7 and ^18^F-rhPSMA-7.3 are similar, but with some slight, but notable differences that are relevant to prostate cancer imaging. Defining the extent of urinary excretion of novel PSMA ligands is imperative given that PSMA ligands that are mainly excreted via the urinary tract often show high radiotracer uptake in the bladder that can interfere with image interpretation in the prostate region [17, 18]. Our data show low relative bladder uptake with both rhPSMA ligands, but, of the two ligands, bladder uptake is significantly lower with ^18^F-rhPSMA-7.3 than ^18^F-rhPSMA-7. Moreover, while the tumour uptake of both rhPSMA ligands is higher than the kidney uptakes, facilitating easy localization of tumours with both rhPSMA ligands via PET/CT, there was a trend towards higher tumour SUV with ^18^F-rhPSMA-7.3. The qualitative PET/CT image interpretation analysis confirm the suitability of both agents for prostate imaging.

Some limitations to the present study should be acknowledged. First, direct conversion of preclinical dosimetry studies to humans may not truly reflect human dosimetry, and thus, interpretation of the estimated effective doses reported here should be performed with caution. Second, our observations of low bladder retention of both ligands may be confounded by the application of furosemide at the time of tracer injection which is routinely done at our institution. It is known from ^68^Ga-PSMA-11 that this can substantially lower bladder retention due to forced bladder emptying prior to the PET scan. Third, we acknowledge a trend towards higher tumour SUV using ^18^F-rhPSMA-7.3 compared with ^18^F-rhPSMA-7. However, an inter- rather than an intra-patient comparison can be affected by several clinical variables (e.g. tumour stage, differences in PSMA-expression, prior treatments)**.** Fourth, definitive conclusions about the uptake characteristics in tumors comparing both cohorts are limited as they base on different patient cohorts. Despite the patient cohorts are weighted for disease stage differences in the distribution of primary Gleason Scores and PSA at imaging have to be acknowledged.

## Conclusion

Radiation dosimetry is favourable both for ^18^F-rhPSMA-7 and ^18^F-rhPSMA-7.3 with an estimated effective dose of less than 5 mSv, assuming a 1 h voiding interval after injection of 370 MBq of the radiotracer. Human biodistribution of ^18^F-rhPSMA-7 and ^18^F-rhPSMA-7.3 were similar to each other, but significantly lower bladder uptake and a trend towards higher uptake in tumour lesions was seen with ^18^F-rhPSMA-7.3.

## Supplementary Information

**Additional file 1**: **Supplementary Table 1.** Mean ± SD %ID/g for ^18^F-rhPSMA-7. Supplementary Table 2 Mean ± SD %ID/g for ^18^F-rhPSMA-7.3. **Supplementary Table 3.** Radiation dose estimates for ^18^F-rhPSMA-7 and ^18^F-rhPSMA-7.3 at 1h and 3.5h bladder voiding intervals. **Supplementary Table 4.** Comparisons of qualitative image parameters between ^18^F-rhPSMA-7 and ^18^F-rhPSMA-7.3 in humans. **Supplementary Table 5.** Clinical characteristics of patients who underwent Supplementary table 5. Clinical characteristics of patients who underwent 18F-rhPSMA-7 (n = 47) and 18F-rhPSMA-7.3 (n = 33) PET/CT.F-rhPSMA-7 (n=47) and Supplementary table 5. Clinical characteristics of patients who underwent 18F-rhPSMA-7 (n=47) and 18F-rhPSMA-7.3 (n = 33) PET/CT.F-rhPSMA-7.3 (n = 33) PET/CT. **Figure 1.** Two examples of patients with early biochemical recurrence (PSA<1 ng/ml) after radical prostatectomy who have undergone ^18^F-rhPSMA-7 (A) and ^18^F-rhPSMA-7.3 PET/CT (B). Images display local recurrences (arrows) in both patients (Patient A shows additional pelvic and retroperitoneal lymph node metastases).

## Data Availability

Anonymised data are available from the Corresponding author on reasonable request.

## Abbreviations

PSMA: Prostate-specific membrane antigen
rhPSMA: Radiohybrid PSMA
SCID: Severe combined immunodeficiency
AUC: Area under curve
%ID: Percentages of the injected dose
PET: Positron emission tomography
CT: Computed tomography
SUV_max_: Maximum standardized uptake value
SUV_mean_: Mean standardized uptake value
VOI: Volume(s) of interest
