# Supplementary material for: Preclinical biodistribution and dosimetry and human biodistribution comparing 18F-rhPSMA-7 and single isomer 18F-rhPSMA-7.3
Source: EJNMMI Res. 2022 Feb 4;12:8. doi: 10.1186/s13550-021-00872-w (PMC8816989; doi:10.1186/s13550-021-00872-w)

**Supplementary Table 1** Mean ± SD %ID/g for ^18^F-rhPSMA-7

|  | **^18^F-rhPSMA-7 in Severe Combined Immunodeficiency Mice** | | | | | |
| --- | --- | --- | --- | --- | --- | --- |
|  | **10 min** | **20 min** | **40 min** | **60 min** | **120 min** | **180 min** |
| **Blood** | 7.16±1.32 | 4.16±1.04 | 1.44±0.29 | 0.67±0.11 | 0.34±0.15 | 0.07±0.03 |
| **Heart*** | 3.96±0.39 | 2.34±0.62 | 0.88±0.29 | 0.45±0.12 | 0.33±0.11 | 0.09±0.03 |
| **Lung*** | 5.19±0.27 | 3.89±0.75 | 1.30±0.22 | 0.90±0.15 | 0.62±0.09 | 0.19±0.04 |
| **Liver*** | 2.25±0.42 | 1.75±0.49 | 0.68±0.21 | 0.46 ±0.23 | 0.35±0.07 | 0.16±0.04 |
| **Spleen*** | 25.85±6.88 | 34.02±9.54 | 18.48±4.99 | 16.32±2.40 | 18.38±7.47 | 3.29±0.80 |
| **Pancreas** | 1.46±0.21 | 1.33±0.25 | 0.44±0.13 | 0.37±0.08 | 0.23±0.04 | 0.08±0.03 |
| **Fat** | 1.49±0.21 | 1.33±0.35 | 1.41±0.58 | 0.57±0.21 | 0.63±0.27 | 0.30±0.18 |
| **Stomach** | 2.35±0.50 | 1.56±0.48 | 0.47±0.11 | 0.35±0.12 | 0.23±0.03 | 0.16±0.12 |
| **Small intestine** | 1.77±0.30 | 1.27±0.30 | 0.47±0.11 | 0.23±0.03 | 0.16±0.03 | 0.08±0.08 |
| **Large intestine** | 2.18±0.26 | 1.29±0.27 | 0.56±0.13 | 0.25±0.04 | 0.21±0.04 | 0.09±0.06 |
| **Kidney*** | 7.73±5.81 | 73.31±33.32 | 19.72±10.30 ^#^ | 44.89±16.18 | 37.34±21.48 | 43.19±8.39 |
| **Muscle** | 1.84±0.36 | 1.02±0.16 | 0.56±0.27 | 0.33±0.13 | 0.22±0.05 | 0.06±0.03 |
| **Femur** | 2.56±0.32 | 2.48±0.30 | 1.16±0.12 | 1.14±0.23 | 1.28±0.39 | 0.85±0.40 |
| **Bladder** | 29.76±37.24 | 10.12±4.20 | 3.95±3.83 | 16.99±10.15 | 12.49±17.30 | 1.32±1.47 |
| **Testicles** | 1.40±0.31 | 2.05±0.29 | 0.85±0.40 | 0.66±0.18 | 1.73±2.10 | 0.16±0.04 |
| **Tail** | 5.08±2.72 | 5.58±1.79 | 1.56±0.17 | 0.90±0.16 | 0.67±0.36 | 0.63±0.27 |
| **Brain** | 0.21±0.03 | 0.19±0.05 | 0.08±0.05 | 0.03±0.01 | 0.03±0.09 | 0.02±0.02 |

*Source organ.

n=5 at all timepoints

^#^ The mean ± SD %ID/g for 18F-rhPSMA-7 at the 40 min timepoint might be biased by measurement errors. However, given the extrapolation of the area-under-the curve this should not have a substantial effect on the further calculations.

**Supplementary Table 2** Mean ± SD %ID/g for ^18^F-rhPSMA-7.3

|  | **^18^F-rhPSMA-7.3 in Severe Combined Immunodeficiency Mice** | | | | | |
| --- | --- | --- | --- | --- | --- | --- |
|  | **10 min** | **60 min** | **120 min** | **180 min** | **300 min** |  |
| **Blood** | 7.29±1.66 | 0.87±0.44 | 0.22±0.13 | 0.15±0.04 | 0.05±0.02 |  |
| **Heart*** | 4.21 ±1.10 | 0.73±0.37 | 0.35±0.23 | 0.17±0.06 | 0.20±0.10 |  |
| **Lung*** | 6.02±1.54 | 1.13±0.40 | 0.55±0.26 | 0.36±0.12 | 0.18±0.05 |  |
| **Liver*** | 2.61±0.54 | 0.50±0.16 | 0.35±0.10 | 0.38±0.09 | 0.31±0.03 |  |
| **Spleen*** | 28.77±10.03 | 20.58±6.17 | 8.24±5.59 | 5.39±3.43 | 1.42±0.54 |  |
| **Pancreas** | 1.49±0.30 | 0.35±0.20 | 0.23±0.16 | 0.14±0.07 | 0.11±0.05 |  |
| **Fat** | 1.97±0.65 | 1.61±0.66 | 1.23±1.29 | 0.51±0.33 | 0.33±0.06 |  |
| **Stomach** | 2.49±0.61 | 0.53±0.13 | 0.33±0.24 | 0.24±0.18 | 0.22±0.08 |  |
| **Small intestine** | 1.85±0.44 | 0.32±0.12 | 0.19±0.11 | 0.14±0.05 | 0.13±0.05 |  |
| **Large intestine** | 2.59±0.65 | 0.34±0.14 | 0.16±0.09 | 0.15±0.04 | 0.19±0.08 |  |
| **Kidney*** | 8.09±5.22 | 21.93±10.58 | 55.73±17.62 | 79.13±17.68 | 44.35±15.87 |  |
| **Muscle** | 1.78±0.45 | 0.56±0.27 | 0.46±0.52 | 0.12±0.04 | 0.10±0.04 |  |
| **Femur** | 2.86±0.77 | 1.50±0.40 | 1.45±0.46 | 1.78±0.66 | 2.09±0.39 |  |
| **Bladder** | 22.97±27.63 | 20.18±19.28 | 10.29±7.43 | 6.81±10.34 | 6.13±3.63 |  |
| **Testicles** | 1.53±0.32 | 1.30±0.69 | 0.52±0.25 | 0.30±0.13 | 0.28±0.08 |  |
| **Tail** | 5.30±2.04 | 1.73±0.48 | 0.74±0.24 | 0.88±0.48 | 0.68±0.12 |  |
| **Brain** | 0.22±0.05 | 0.06±0.04 | 0.03±0.01 | 0.03±0.01 | 0.02±0.01 |  |

*Source organ.

n=6 at all timepoints, except for 300 min (n=3).

**Supplementary Table 3** Radiation dose estimates for ^18^F-rhPSMA-7 and ^18^F-rhPSMA-7.3 at 1h and 3.5h bladder voiding intervals

|  | ^18^F-rhPSMA-7 | | ^18^F-rhPSMA-7.3 | |
| --- | --- | --- | --- | --- |
| Target Organ | Absorbed dose at 1h  (µGy/MBq) | Absorbed dose at 3.5h  (µGy/MBq) | Absorbed dose at 1h  (µGy/MBq) | Absorbed dose at 3.5h  (µGy/MBq) |
| Adrenals | 7.59 | 7.80 | 10.90 | 11.00 |
| Brain | 4.49 | 4.49 | 7.18 | 7.19 |
| Breasts | 4.20 | 4.24 | 6.65 | 6.67 |
| Gallbladder wall | 6.91 | 7.49 | 10.20 | 10.60 |
| LLI wall | 9.20 | 16.10 | 11.60 | 15.90 |
| Small intestine | 7.73 | 10.40 | 10.90 | 12.50 |
| Stomach wall | 6.65 | 7.00 | 9.92 | 10.10 |
| ULI wall | 7.27 | 9.26 | 10.50 | 11.70 |
| Heart wall | 4.36 | 4.42 | 6.68 | 6.71 |
| Kidneys | 64.70 | 65.10 | 71.60 | 71.80 |
| Liver | 4.02 | 4.27 | 5.84 | 5.99 |
| Lungs | 4.26 | 4.30 | 6.58 | 6.61 |
| Muscle | 5.96 | 7.68 | 8.59 | 9.67 |
| Ovaries | 9.16 | 15.50 | 11.70 | 15.70 |
| Pancreas | 7.80 | 8.08 | 11.20 | 11.40 |
| Red marrow | 5.68 | 6.80 | 8.31 | 9.01 |
| Osteogenic cells | 8.27 | 8.93 | 12.80 | 13.20 |
| Skin | 4.32 | 4.93 | 6.57 | 6.95 |
| Spleen | 25.80 | 26.10 | 26.60 | 26.70 |
| Testes | 7.03 | 11.60 | 9.20 | 12.10 |
| Thymus | 5.15 | 5.18 | 8.18 | 8.20 |
| Thyroid | 5.17 | 5.18 | 8.27 | 8.27 |
| Urinary bladder wall | 115 | 354 | 78.40 | 227 |
| Uterus | 13.10 | 28.00 | 14.30 | 23.60 |
| Total body | 6.19 | 7.77 | 8.87 | 9.86 |

LLI: lower large intestine; ULI: upper large intestine.

**Supplementary Table 4** Comparisons of qualitative image parameters between ^18^F-rhPSMA-7 and ^18^F-rhPSMA-7.3 in humans

|  |  | ^18^F-rhPSMA-7 | ^18^F-PSMA-7.3 | *p* value |
| --- | --- | --- | --- | --- |
|  |  |  |  |  |
| Overall subjective image quality (1-4) | 1 (Good) | 44.7% (n=21) | 57.6% (n=19) | 0.4819 |
|  | 2 (Moderate) | 42.6% (n=20) | 36.4% (n=12) |  |
|  | 3 (Poor) | 12.8% (n=6) | 6.1% (n=2) |  |
|  | 4 (non-interpretable) | 0% (n=0) | 0% (n=0) |  |
| Nonspecific blood pool activity  (1-3) | 1 (No) | 8.5% (n=4) | 3.0% (n=1) | 0.0631 |
|  | 2 (Slight, mainly central vessels) | 80.9% (n=38) | 66.7% (n=22) |  |
|  | 3 (Moderate) | 10.6% (n=5) | 30.3% (n=10) |  |
| Background uptake in bone/marrow  (1-4) | 1 (No) | 0% (n=0) | 0% (n=0) | 0.6160 |
|  | 2 (Slight (<gluteal muscle) | 58.7% (n=27) | 71.0% (n=22) |  |
|  | 3 (Moderate (>gluteal muscle)) | 32.6% (n=15) | 22.6% (n=7) |  |
|  | 4 (Focal Spots) | 8.7% (n=4) | 6.5% (n=2) |  |
| Negative impact of biodistribution on clinical decision making (Yes/No) | Yes | 8.5% (n=4) | 6.1 (n=2) | 1.0000 |

Image quality was analyzed using scales reported previously [7].

**Supplementary table 5.** Clinical characteristics of patients who underwent ^18^F-rhPSMA-7 (n=47) and ^18^F-rhPSMA-7.3 (n=33) PET/CT.

|  | Prostate cancer stage | Gleason Score |
| --- | --- | --- |
| ^18^F-rhPSMA-7 (n=47) | Primary staging: n=5  Biochemical recurrence: n=42 | Gleason Score 6: n=4  Gleason Score 7a: n=1  Gleason Score 7b: n=4  Gleason Score 8: n=6  Gleason Score 9: n=14  Gleason Score 10: n=1  unknown: n=17 |
| ^18^F-rhPSMA-7.3 (n=33) | Primary staging: n=3  Biochemical recurrence: n=30 | Gleason Score 6: n=1  Gleason Score 7a: n=5  Gleason Score 7b: n=5  Gleason Score 8: n=6  Gleason Score 9: n=6  Gleason Score 10: n=0  unknown: n=7 |

**Supplementary Figure 1.** Two examples of patients with early biochemical recurrence (PSA<1 ng/ml) after radical prostatectomy who have undergone ^18^F-rhPSMA-7 (A) and ^18^F-rhPSMA-7.3 PET/CT (B). Images display local recurrences (arrows) in both patients (Patient A shows additional pelvic and retroperitoneal lymph node metastases).


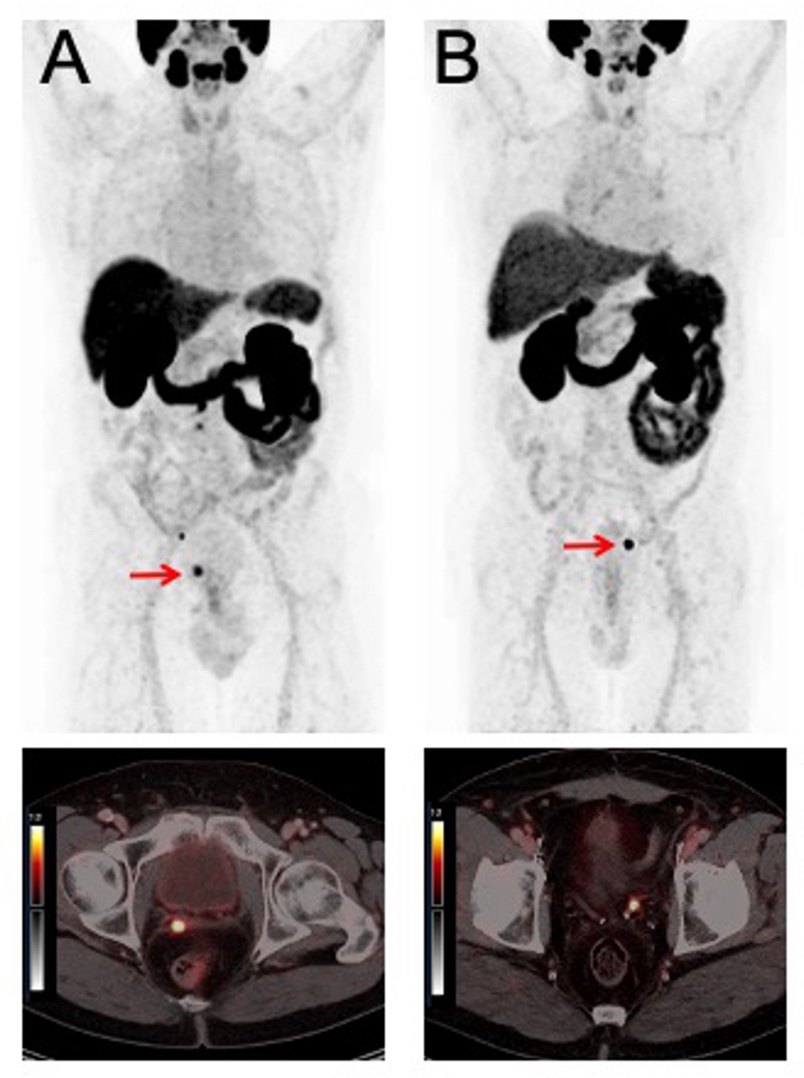

Supplement: Supplementary file 1 — Additional file 1: Supplementary Table 1. Mean ± SD %ID/g for 18F-rhPSMA-7. Supplementary Table 2 Mean ± SD %ID/g for 18F-rhPSMA-7.3. Supplementary Table 3. Radiation dose estimates for 18F-rhPSMA-7 and 18F-rhPSMA-7.3 at 1h and 3.5h bladder voiding intervals. Supplementary Table 4. Comparisons of qualitative image parameters between 18F-rhPSMA-7 and 18F-rhPSMA-7.3 in humans. Supplementary Table 5. Clinical characteristics of patients who underwent Supplementary table 5. Clinical characteristics of patients who underwent 18F-rhPSMA-7 (n = 47) and 18F-rhPSMA-7.3 (n = 33) PET/CT.F-rhPSMA-7 (n=47) and Supplementary table 5. Clinical characteristics of patients who underwent 18F-rhPSMA-7 (n=47) and 18F-rhPSMA-7.3 (n = 33) PET/CT.F-rhPSMA-7.3 (n = 33) PET/CT. Figure 1. Two examples of patients with early biochemical recurrence (PSA<1 ng/ml) after radical prostatectomy who have undergone 18F-rhPSMA-7 (A) and 18F-rhPSMA-7.3 PET/CT (B). Images display local recurrences (arrows) in both patients (Patient A shows additional pelvic and retroperitoneal lymph node metastases). [file 13550_2021_872_MOESM1_ESM.docx]
